# Progress in Surgery

**Published:** 1898-01-01

**Authors:** 


### PROGRESS IN SURGERY.

### GENERAL SURGERY.

(*Continued from page 226.*)

**Fractures and Dislocations.—Upper Extremity.** — In order to avoid the functional impotence so often met with in cases of recent and uncomplicated dislocation of the shoulder, treated by strict immobilisation according to the ordinary method, Dr. A. Massy[23] has had recourse, with good result, to massage of the dislocated joint, performed immediately after reduction. After reduction has been effected, the arm is fixed against the thorax so that the elbow is carried and kept in the same direction as the dislocation itself, and the limb fixed in Mayor's four-headed sling, or a simple spiral bandage round the arm and chest may be used. Two hours afterwards massage is commenced and continued in increasing extensiveness daily until pain is no longer produced by digital pressure or by active movement of the joint, and until the various movements of the joint have recovered their normal amplitude. In Dr. Massy's cases a complete cure resulted within three to four weeks. The pathology, etiology, and treatment of habitual or recurrent dislocation of the shoulder is the subject of a concise account by Dr. H. L. Burrell and R. W. Lovett.[24] The former surgeon, through an anterior incision, opened the joint, explored it, removed an elliptical piece of the capsule, and sutured the capsule, thereby shortening it. The account of this case is given in detail. He operated on a second case, excising three-quarters of an inch of the capsule in front. The important steps of the operation are: The free division of the tendinous insertion of the pectoralis major for three-fourths of its breadth, in order that the head of the bone and capsular ligament may be freely exposed by retracting the muscle; the division of a portion of the insertion of the subscapularis; raising the arm to a horizontal plane, and pressing back the head of the bone, which relaxes the front of the capsule so that it can be grasped, and a portion excised. A figure of a dissection to demonstrate this procedure is also given. The results of operative treatment of irreducible dislocation of the shoulder, whether recent or old, simple or complicated with fracture, are discussed in a recent paper by Dr. Edmond Souchon,[25] based on 140 cases. In all recent irreducible dislocations operation by reduction or resection has given good results, except in two, in which death ensued, apparently from shock. Old, irreducible, simple, and forward dislocations, treated by resection through an anterior incision, were the most frequent—56 cases in all, as against 33 treated by arthrotomy and reduction. The remote results show great mortality, in cases of resection, from injuries to vessels. A fatal issue in reduction is due to sepsis, which is now preventable. Reduction is a more desirable operation than resection, because it preserves the head, and all movements depending thereon; but the necrotic consequences in the cartilage and bone, and ankylosis, which sometimes follows the reduction, are serious drawbacks. A subcoracoid dislocation was operated on by Professor C. Fenger[26] five weeks after accident. After attempts at reduction by Kocher's method a vertical incision outside the coracoid process was made, and the head of the humerus exposed by opening the capsule. It was found that the anterior part of the greater tuberosity was broken off. This and the adjacent portion of the capsule was therefore removed, and the humeral head reduced. The patient made a good recovery. Dr. G. R. Saunders[27] has lately operated successfully upon a case of ununited fracture of the shaft of the humerus nearly sixteen months after the accident. After incision of the soft parts the ends of the humerus were thoroughly freshened and screwed together. The screws were removed about the twenty-first day, and the patient was finally able to use his arm for manual labour as well as before the accident. Professor Tillaux[28] records a successful example of operation for pseudarthrosis of the same bone. The surfaces of the ends were freshened, pegged, and sutured with silver wire; in addition, the parts were covered with a flap of periosteum from the femur of a dog. Consolidation took place. Aikins' hoop iron splint, in fractures of the humerus, described by George A. Peters,[29] is at once, in everyday practice, a simple, effectual, and valuable method of making extension in all fractures of the shaft or of the upper or lower end of this bone. The material is the ordinary flexible band or hoop iron, procurable at any ironmonger's or blacksmith's shop. For infants and very young children a band 1 in. wide is amply strong; for adults a width of 1¼ to 2 in. is of sufficient strength. The thickness and strength of the material are usually in proportion to the width. It may be bent by hand or over the edge of a table or by a pair of wrenches. This, having been selected according to the muscularity of the subject, is applied to the shoulder as follows: It is bent and twisted in such a manner that an arch is formed over the top of the shoulder. The anterior limb of this arch reaches downwards over the front of the chest in a direction somewhat towards the umbilicus, while the posterior limb forms the vertical part of the splint down the back of arm to below the elbow, thence on to the posterior aspect of the forearm in a direction towards the mid-line of the body. The splint almost forms a triangle, and it is not necessary or convenient that it should fit the shoulder or elbow accurately on account of the pressure on bony prominences. A distinctive feature of the splint is that the distance from the shoulder-arch to the bend under the elbow should be such that when the arch is fixed in position by strapping, considerable extension may be made on the muscles of the arm by drawing the forearm down to the horizontal line of the splint below the elbow. It may be padded to any desired extent by wrapping it with a roller of sheet cotton. Dr. A. B. Johnson[30] records a rare case of avulsion of the biceps tendon, from the radius in which he cut down upon and trimmed the end, and sutured it to the periosteum round the tuberosity. The result was very satisfactory. In the treatment of Colles's fracture of the radius Dr. E. A. Tracy[31] is of opinion that passive motion is important at the earliest possible time, and that immobilisation is not indicated. He recommends the use of wood-fibre splints, which can be removed for the purpose of motion. It permits of the introduction of antisepsis, and is also pervious to the X rays. He condemns plaster of Paris as dangerous, and a thing of the past. For the correction of deformity supposed to be due to Colles's fracture, in a boy of about 16, one and a half inches from the lower end, Dr. McBurney[32] cut down upon the bone, divided it close to the line of fracture, and replaced the parts in proper position and applied a plaster of Paris splint. Very little deformity resulted, and the restoration of function was extremely satisfactory. Dr. J. B. Roberts'[33] description of fracture of the radius with forward displacement of the lower fragment, described in THE HOSPITAL of April 3rd, 1897, has been published in the form of a complete monograph on the subject, and should be in every surgeon's library. Kramer[34] records a rare case of compound separation of the lower radial epiphysis, complicated with compound dislocation of the elbow backwards on the same side. The median nerve was partially torn through by the end of the humerus being displaced forwards. The dislocation was reduced, the nerve sutured, and the radial epiphysis replaced. An interrupted recovery took place with considerable restoration of the functions of the wrist and elbow. Dr. E. R. Corson[35] gives radiographs of four wrists diagnosed and treated as Colles's fracture. In one instance, in which a surgeon informed the patient that the bone should be refractured and reset, the radiograph showed that the radius was in fairly good position: there being but slight dorsal displacement of the lower fragment. Had an attempt been made to rebreak the bone, matters would have been made worse. The mistake made was ascribing the deformity to the bones when it was the ruptured ligaments which were the cause of the trouble. Corson thinks that there should be a new treatise on fractures and dislocations based on the X ray. Dr. E. H. Bennett[36] has now fully confirmed the fact that fracture of the base of the metacarpal bone of the thumb is the commonest metacarpal fracture. With the X rays the photographs established the details of the recent injury and corroborate the view he had published as to the path of the fracture passing obliquely through the bone without implication of the dorsal surface of the bone. His photographs, taken by Dr. W. S. Haughton from two patients, one a three days old fracture, the second three weeks after the injury, and his casts of hands and X ray photographs from a man who had sustained the injuries in both hands simultaneously by a fall from a ladder, the deformities in both hands being characteristic, all go to prove the value of these investigations and to indicate that the injury in the future should be associated with the name of Professor Bennett. Mr. E. W. Roughton[37] produces a skiagraph of a peculiar case of congenital shortness of metacarpal bones associated with a like condition of the metatarsus. Although the patient attributed the deformity of the hands to an injury which she had received in childhood, she could offer no explanation of the condition of the feet. He thought that the deformity was of congenital origin, but was unable to offer any further explanation.

[23] Med. Week, June 4, 1897. [24] The Amer. Journ. of the Med. Sciences, August, 1897, p. 166. [25] N.Y. Med. Record, June 5, 1897, p. 818; and Medical Week, June 4, 1897, p. 255. [26] Chicago Clinical Review, Sept., 1897, p. 627. [27] Australasian Medical Gazette, June 22, 1897, p. 265, [28] Medical Week, July 9, 1897. [29] Brit. Med. Journ., June 5, 1897, p. 1,409. [30] N.Y. Med. Jour., August 21, 1897, p. 261. [31] N. Y. Med. Record, July 17, 1897. [32] N. Y Med. Record, April 3, 1897, p. 495. [33] Fractures of the Radius, Philadelphia, 1897. [34] Monatsschrift für Unfallheilkunde, 1897, No. 4. [35] N.Y. Medical Record, May 8, 1897, p. 649. [36] Brit. Med. Jour., June 12, 1897. [37] Lancet, July 3, 1897, p. 19.

